# Interleukin Concentrations in Bone Marrow Fluid and MRI Prognostic Findings in Osteoporotic Vertebral Fractures

**DOI:** 10.7759/cureus.93562

**Published:** 2025-09-30

**Authors:** Yasuhiro Nakajima, Akinori Kageyama, Yasukazu Hijikata, Ayako Motomura, Takashi Tsujiuchi, Koji Osuka

**Affiliations:** 1 Department of Neurosurgery, Daido Hospital, Nagoya, JPN; 2 Department of Neurosurgery, Nagoya University Hospital, Nagoya, JPN; 3 Section of Clinical Epidemiology, Department of Community Medicine, Kyoto University, Kyoto, JPN; 4 Department of Neurosurgery, Aichi Medical University, Nagakute, JPN

**Keywords:** bone marrow aspirate, interleukin, poor prognosis, vertebral fracture, vertebroplasty

## Abstract

Objective

We investigated the association between interleukin-6 (IL-6) and interleukin-8 (IL-8) concentrations in bone marrow fluid and osteoporotic vertebral fracture (OVF) prognosis to obtain insights that could improve the prediction of poor prognosis.

Methods

Patients with OVFs admitted consecutively to a single facility and who underwent balloon kyphoplasty were prospectively enrolled. Bone marrow fluid was collected during surgery, and IL-6 and IL-8 concentrations were measured. Independent experts blinded to patient details examined the preoperative magnetic resonance imaging (MRI) radiographs to identify poor prognostic findings. IL-6 and IL-8 levels in the groups with and without poor prognostic findings were compared using the Wilcoxon rank-sum test. Associations between log-transformed IL-6 and IL-8 levels and poor prognosis were evaluated using logistic regression with adjustments for confounding factors.

Results

Among the 95 enrolled patients, 49 were excluded, and 46 were included in the analysis. In the poor prognosis group, both IL-6 and IL-8 levels were higher than those in the non-poor prognosis group (median IL-6: 491 vs. 72 pg/mL, p=0.014; median IL-8: 1574 vs. 84 pg/mL, p=0.003). Associations between log-transformed IL-6 and IL-8 concentrations and poor prognosis were observed, with crude and adjusted odds ratios for IL-6 at 1.75 (95% confidence interval (CI) 1.10-2.81) and 1.95 (95% CI 1.05-3.63), respectively, and for IL-8 at 1.81 (95% CI 1.23-2.65) and 2.10 (95% CI 1.21-3.64), respectively.

Conclusion

IL-6 and IL-8 concentrations in the bone marrow fluid may be associated with poor OVF prognosis. Their causal roles and potential as serum prognostic markers require further investigation.

## Introduction

In the aging societies of East Asian countries, the incidence of osteoporotic vertebral fractures (OVFs) in older individuals is very high, and some patients with OVFs have significantly reduced quality of life due to pain caused by kyphotic deformity of the spine and neuropathy caused by nerve compression from deformation or compression of the fractured vertebra [1]. Percutaneous vertebroplasty (PVP) is a treatment option for OVF but has been ruled out as the standard treatment [2,3]. PVP and balloon kyphoplasty (BKP) can reduce the quality of life of patients by limiting the target population for treatment [4,5]. Although it has been established that PVP should be indicated for patients with OVFs with poor prognosis, the prognostic factors impacting OVF outcomes are not well understood [6-10]. Therefore, identifying prognostic factors relevant to OVFs is important.

Several studies have indicated an association between OVFs and inflammatory cytokines [11-16]. Increased inflammatory cytokine levels have been observed during osteoporosis progression and have been reported to lead to bone fragility and an elevated risk of vertebral body fractures [17]. Interleukin-6 (IL-6) and interleukin-8 (IL-8) are recognized as inflammatory cytokines that promote bone resorption and prevent bone formation [18-20]. Vertebral fractures are usually associated with local bone damage and inflammation, and IL-6 concentrations in bone marrow fluid at the fracture site tend to increase during the acute phase of fracture. High IL-6 and IL-8 concentrations in the acute phase of fractures can promote bone resorption and simultaneously interfere with bone formation, leading to prolonged healing and fractures refractory to conservative treatment.

Therefore, in this study, as a first step in establishing that high concentrations of IL-6 and IL-8 in bone marrow fluid contribute to the poor prognosis of OVF, we first verified that IL-6 and IL-8 concentrations in bone marrow fluid collected from fractured vertebrae during BKP are associated with a poor prognosis of OVFs.

## Materials and methods

Study design and participants

We used a cross-sectional design and prospectively collected data. The study was conducted in accordance with the Declaration of Helsinki [21] and the STROBE Statement [22] and was approved by the relevant Ethics Committee (Ethical Approval Number: ECD2020-015). Written informed consent was obtained from all patients. Consecutive patients with OVFs who underwent BKP performed at our institution between July 2020 and April 2023 were included in this study. We excluded patients with vertebral infections such as pyogenic spondylitis, pathological vertebral fractures due to metastatic vertebral tumors or multiple myeloma, symptomatic spinal canal stenosis, patients with prior surgery on the fractured vertebra, and patients for whom a sufficient volume of bone marrow fluid could not be collected intraoperatively to allow the measurement of cytokine concentrations.

Exposure variable 

Bone marrow fluid was collected intraoperatively via an osteo-introducer during BKP. Under fluoroscopic guidance, an 8G (3.2-mm diameter) osteo-introducer was inserted into the vertebral body via a transpedicular approach. Only the outer cannula of the introducer was left within the vertebral body, through which the balloon was subsequently inserted. Inflation of the balloon caused a small amount of bone marrow fluid to leak out through the outer cannula. This fluid was aspirated by an assistant using a syringe with an 18G needle and immediately centrifuged, and the supernatant was frozen for cytokine concentration measurement. Enzyme-linked immunosorbent assay (ELISA) kits were used to measure the concentrations of IL-6 (IL-6 Human Uncoated ELISA Kit, Cat #88-7066-22; EIA; Invitrogen, Inc.) and IL-8 (IL-8 Human Uncoated ELISA Kit, Cat #88-8086-22; EIA Invitrogen, Inc.). The detection limit of the ELISA was 2 pg/mL for IL-6 and IL-8.

Outcome

**Figure 1:**
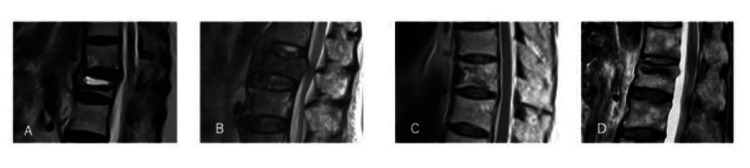
Four types of MRI T2WI classification (A) Locally confined high intensity; (B) Diffuse low intensity; (C) Mosaic; (D) Other lesions.

We evaluated the radiographic attributes indicating poor prognosis for vertebral fractures on magnetic resonance imaging (MRI) (T2-weighted images (T2WI)) at the time of diagnosis. MRI findings from the evaluation of T2WI were classified into one of four types: locally confined high intensity, diffuse low intensity, mosaic, or other lesions (Figure 1). The diagnostic classification was made independently by two certified spine surgeons who were blinded to the IL-6 and IL-8 bone marrow fluid concentrations linked to the MRI radiographs. The advice of a certified radiologist was sought in cases of disagreement. Inter-rater agreement among the three raters was assessed using the Fleiss’ kappa coefficient. In the event of further discrepancies, a certified radiologist was consulted. Based on the findings of previous studies, locally confined high-intensity and diffuse low-intensity lesions were defined as poor prognostic findings [23,24].

Covariates

The following background information was collected: age, sex, affected vertebral level, preoperative pain (numeric rating scale (NRS)), intervertebral cleft, preoperative kyphotic angle, days from injury to MRI, days from injury to surgery, days from MRI to surgery, and bone density. The preoperative kyphosis angle was defined as the angle between the upper and lower endplates of the fractured vertebra on a standing radiograph, and kyphosis was defined as a positive angle. Bone density was assessed using coronal sections of images obtained via three-dimensional reconstructive spine computed tomography (CT) (tube voltage, 120 kV; Aquilion ONE, TOSHIBA), with CT values recorded at the center of the vertebral body (in Hounsfield Units). The L1 vertebra was selected for measurement; however, if the L1 vertebra was fractured, the L2 vertebra was used. If both were fractured, the CT values of the T12 vertebra were used as an alternative.

Statistical analysis

Baseline characteristics were described for the entire patient cohort as well as the presence or absence of poor prognostic MRI findings. Data were summarized using the median and interquartile range (IQR) for continuous variables and number and frequency for categorical variables. To measure the differences in baseline characteristics, p-values were calculated using Wilcoxon's rank-sum test for continuous variables and Fisher's exact test for categorical variables. Histograms showing the distribution of IL-6 and IL-8 levels, with and without poor prognostic findings, were also created. Logistic regression was used, with poor prognosis as the objective variable and IL-6 and IL-8 concentrations as explanatory variables. Odds ratios and 95% confidence intervals (CIs) were calculated. Age, sex, preoperative kyphosis, days to MRI, and CT values were included as confounding factors in the model, and IL-6 and IL-8 levels and days to MRI were log-transformed for analysis. STATA 17.0 software (Stata Corp., College Station, TX, USA) was used for the analysis.

## Results

**Figure 2:**
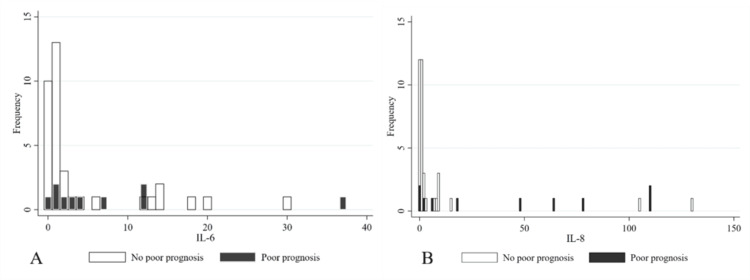
Histograms of IL-6 and IL-8 concentrations in patients with and without poor prognostic findings in MRI a: IL-6, b: IL-8. Black-shaded bars: group with poor prognostic findings; white-shaded bars: group without poor prognostic findings. The horizontal axis is in pg/mL/100. The vertical axis represents frequency (%).

There were 95 patients who were enrolled in this study. However, three of these patients were excluded because of suspected metastatic vertebral tumors, and 46 were excluded because it was not possible to obtain a sufficient volume of intraoperative bone marrow fluid from them to measure cytokine concentrations. Thus, the total number of participants was 46. There were no missing variables in this study. The median patient age was 80 years and 30 patients (65%) were female (Table 1). The preoperative NRS score was high, with a median of 9.5 (IQR 8.9, 9.8). The median time from injury to diagnosis was seven days. Comparing the poor prognosis and non-poor prognosis groups, there were more clefts in the fractured vertebrae of the poor prognosis group (67% vs. 16%), longer time to diagnosis (18 vs. 5 days), longer time to surgery (35.5 vs. 17 days, respectively), and slightly longer time from diagnosis to surgery (14.5 vs. 10 days, respectively). Inter-rater agreement among the three raters was moderate (Fleiss’ kappa coefficient = 0.64 (95%CI 0.49, 0.79)). Both IL-6 and IL-8 concentrations were higher in the poor prognosis group than in the non-poor prognosis group (IL-6: 491 vs. 72, p=0.014; IL-8: 1574 vs. 84, p=0.003). The distribution of IL-6 and IL-8 levels in patients with and without poor prognosis is shown in Figure 2. A trend toward high IL-8 concentrations was observed in the poor-prognosis group.

**Table 1:** Baseline characteristics of study participants Data are presented as number (%) or median (interquartile range). IL-6, interleukin-6; IL-8, interleukin-8; MRI, magnetic resonance imaging;NRS, Numeric Rating Scale; HU, Hounsfield Units.

	Total	Poor prognosis	No poor prognosis	p-value
	n = 46	n = 12	n = 34	
Age, years	80 (77, 84)	81.5 (79, 83)	79.5 (76, 86)	0.95
Female	30 (65)	9 (75)	21 (62)	0.5
Level of the affected vertebra				0.24
Thoracic (T8-T10)	5 (11)	0	5 (15)	
Thoracolumbar junction (T11-L1)	25 (54)	9 (75)	16 (47)	
Lumbar (L2-L5)	16 (35)	3 (25)	13 (38)	
Preoperative NRS	9.5 (8.9, 9.8)	8 (67)	9.5 (9, 9.7)	0.59
Bone density, HU	77 (44, 113)	86 (38, 122)	72 (46, 104)	0.58
Preoperative kyphosis angle	11 (6, 16)	15 (2, 20)	10 (6, 15)	0.19
Intervertebral cleft	17 (37)	8 (67)	9 (26)	0.019
Days from injury to MRI	7 (5, 17)	18 (12, 35)	5 (4, 10)	0.001
Days from injury to surgery	20 (12, 31)	36 (22, 76)	17 (11, 22)	<0.001
Days from MRI to surgery	11 (6, 16)	15 (10, 43)	10 (5, 15)	0.042
IL-6 (pg/mL)	113 (50, 588)	491 (148, 199)	71 (42, 192)	0.014
IL-8 (pg/mL)	117 (41, 920)	1574 (337, 7090)	84 (35, 197)	0.003

The association between log-transformed IL-6 levels and poor prognosis, relative to the group without poor prognosis, was indicated by a crude odds ratio of 1.75 (95% CI 1.10, 2.81) and an adjusted odds ratio of 1.95 (95% CI 1.05, 3.63). Similarly, an association between log-transformed IL-8 concentrations and poor prognosis was suggested, with a crude odds ratio of 1.81 (95% CI 1.23, 2.65) and an adjusted odds ratio of 2.10 (95% CI 1.21, 3.64).

## Discussion

In 46 patients with OVFs, IL-6 and IL-8 concentrations in the bone marrow fluid collected during BKP were associated with poor prognostic MRI findings. This suggests that IL-6 and IL-8 levels contribute to the poor prognosis of vertebral fractures. However, it is not possible to assert that anti-inflammatory treatment improves fracture prognosis based on the results of this study alone because of the limitations of this study, as described below. Nevertheless, the results of this study will motivate more robust studies to explore causal inferences between IL-6/-8 concentrations and poor prognosis in OVF. The results of this study suggest that serum IL-6 and IL-8 concentrations may be predictors of vertebral fracture severity and, ultimately, prognosis. The measurement of cytokine concentrations in the bone marrow fluid is not easily performed in routine practice because it is not a minimally invasive procedure. However, a strong correlation between serum and bone marrow fluid cytokine concentrations, such as IL-6 and IL-8, has been previously reported [25].

Tsujio et al. identified prognostic factors of vertebral fractures in a prospective multicenter study [23]. Acute risk factors for OVF, which are prone to nonunion with conservative treatment, include a vertebral fracture in the thoracolumbar spine, the presence of a middle column injury, and a locally confined high-intensity or diffuse low-intensity area in the fractured vertebrae on T2-weighted MR images. Although several other studies have cited acute risk factors, no well-established prognostic factors for OVFs are known [24]. Therefore, it is important to establish prognostic factors for OVFs. We suggest that IL-6 and IL-8 concentrations in the bone marrow fluid as well as serum IL-6 and IL-8 concentrations, which correlate well with bone marrow fluid concentrations and require less invasive sampling, may be useful prognostic factors for OVFs.

In a previous study, an increase in inflammatory cytokine levels was reported to lead to bone fragility and an elevated risk of vertebral fractures [17]. IL-6, one of the most representative inflammatory cytokines, has been observed to increase in blood concentrations in patients with osteoporosis and is known to promote bone resorption and simultaneously interfere with bone formation [18-20]. Acute vertebral fractures are usually accompanied by local bone damage and inflammation, resulting in increased IL-6 concentrations in the bone marrow fluid near the fracture site. During the acute phase of a vertebral fracture, blood IL-6 concentration increases as a systemic inflammatory response. IL-6 and IL-8 concentrations at the fracture site gradually decreased after the acute phase of the fracture while continuing to be involved in the repair and regeneration processes at the fracture site. High IL-6 and IL-8 concentrations in the acute phase of fracture may promote bone resorption and simultaneously interfere with bone formation, leading to prolonged healing of the fractured vertebrae and induction of vertebral fractures that are highly resistant to conservative treatment.

This study had several limitations. First, this study employed a cross-sectional design, and IL-6/-8 concentrations were measured after the MRI findings (the outcome variable) were determined. Therefore, there is a possibility of reverse causation, and the results should be interpreted as associations rather than causal relationships. Second, IL-6/-8 concentrations are variables that can change over time, which may introduce bias into the estimation. Notably, there was a difference of approximately 19 days between the time from injury to IL-6/-8 concentration measurement in the group with poor prognostic findings and the group without. Therefore, the measured IL-6/-8 concentrations may reflect the disease stage. A previous study reported that serum IL-6 concentrations collected 2-3 weeks after the onset of acute myocardial infarction impaired bone marrow-derived colony capacity in a dose-dependent manner [25]. Based on our study, we hypothesized that IL-6/-8 concentrations in the bone marrow fluid of affected vertebrae have a longer-lasting effect than those in the blood; however, further validation is necessary to confirm the validity of this assumption. Third, there is a risk of selection bias because 49 patients (52%) were excluded due to insufficient bone marrow fluid. This may reflect technical issues or impaired marrow function that could affect prognosis. Thus, our findings apply only to patients from whom sufficient fluid could be obtained. Finally, there is the problem of outcome misclassification. As all study participants had undergone BKP, it was not possible to observe their prognosis longitudinally, including the natural history of OVF. Therefore, we analyzed MRI findings at diagnosis as a surrogate variable for poor prognosis. The MRI findings were evaluated by experts who were completely blinded to the IL-6/-8 concentrations and diagnosed based on the opinions of multiple experts. However, misclassification of outcomes is inevitable. Moreover, the poor prognostic MRI findings in this study represent surrogate markers rather than actual clinical outcomes such as pain, fracture healing, or re-fracture. Longitudinal follow-up studies in patients treated with BKP are still warranted. Moreover, the poor prognostic MRI findings in this study represent surrogate markers rather than actual clinical outcomes such as pain, fracture healing, or re-fracture. Longitudinal follow-up studies in patients treated with BKP are still warranted.

## Conclusions

This study suggests that IL-6 and IL-8 concentrations in the bone marrow fluid may be associated with the poor prognosis of OVFs. These findings are important for more robust studies examining the causal effects of IL-6 and IL-8 on the prognosis of OVFs and for studies examining serum IL-6 and IL-8 levels as prognostic factors for OVFs.
